# The influence of intrathecal baclofen pumps on outcomes following spinal fusion in non-ambulatory patients with cerebral palsy

**DOI:** 10.1007/s43390-026-01274-5

**Published:** 2026-01-17

**Authors:** Lexi M. Larson, Daniel J. Miller, Luis Torres-Gonzalez, Tenner J. Guillaume, Walter H. Truong, Joseph H. Perra, Linda E. Krach, Maykala J. Williams, Sara J. Morgan

**Affiliations:** 1 Research Department, Gillette Children’s, 200 University Ave E, Internal Zip 490105, St. Paul, MN 55101 USA; 2 Rocky Vista University School of Osteopathic Medicine, Parker, CO USA; 3 Department of Orthopaedic Surgery, Gillette Children’s, St. Paul, MN USA; 4 Department of Orthopaedic Surgery, University of Minnesota, Minneapolis, MN USA; 5 University of Illinois College of Medicine, Chicago, IL USA; 6 Twin Cities Spine Center, Minneapolis, MN USA; 7 Department of Rehabilitation Medicine, University of Minnesota, Minneapolis, MN USA; 8 Department of Family Medicine and Community Health, University of Minnesota, Minneapolis, MN USA; 9 Department of Rehabilitation Medicine, University of Washington, Seattle, WA USA

**Keywords:** Neuromuscular scoliosis, Spine surgery, Intrathecal baclofen pumps, Surgical site infection, Pediatrics

## Abstract

**Purpose:**

Children with non-ambulatory cerebral palsy (CP) often have neuromuscular scoliosis (NMS) that requires surgical correction. Many of these children also have co-existing spasticity treated with an intrathecal baclofen pump (ITBP). Theoretically, ITBPs can complicate spine surgery due to the proximity of the pump and catheter to the surgical area, but the evidence on effects of ITBP on spinal deformity surgery outcomes is varied. The aim of this study was to compare surgical outcomes and incidence of complications between children with and without ITBPs.

**Methods:**

This retrospective study included children with CP from a single center who underwent spinal fusion between 2001 and 2021. Complications and outcomes were abstracted from the medical record and compared between those with and without ITBP using Fisher’s exact tests or Mann–Whitney *U* tests.

**Results:**

A total of 334 patients were eligible (ITBP: *n* = 163; no ITBP: *n* = 171). In general, children with ITBP were not more likely to experience complications compared to those without (*p* = 0.19). However, those with ITBP had greater odds of surgical site infection (OR 3.11, *p* = 0.03), longer surgery duration (*p* < 0.001), and higher percentage of blood loss (*p* = 0.01). ITBP-related complications occurred in 11% of children with ITBP.

**Conclusions:**

The presence of ITBP did not increase the general risk of complications for children with ITBP. However, children with ITBP experienced more surgical site infections, longer surgery durations, and a higher percentage of blood loss. Results will improve counseling between surgeons, children, and caregivers regarding the risk of spinal fusion surgery when ITBPs are present.

**Supplementary Information:**

The online version contains supplementary material available at 10.1007/s43390-026-01274-5.

## Introduction

Neuromuscular scoliosis (NMS) occurs in approximately 20 to 25% of children with cerebral palsy (CP) [1]. Non-ambulatory children with NMS are at a significantly higher risk of developing severe, progressive scoliosis (50–100%) [2, 3] which can cause seating intolerance, pain, and pulmonary insufficiency. Surgical intervention is often recommended to prevent these effects [1, 4, 5]; however, comorbidities such as poor nutritional status, antiepileptic medications, and compromised pulmonary function [1] can lead to higher rates of infections, pseudoarthrosis, and pulmonary events in this population [1, 6].

Children with CP also commonly have mixed tone, including spasticity. Severe spasticity is often associated with spasms, pain, and stiffness [7, 8], and is frequently treated with baclofen [8]. Baclofen acts as a gamma-aminobutyric acid (GABA) agonist targeting receptors in the central nervous system. The exact mechanism of action is unknown, but it is speculated to inhibit the release of excitatory neurotransmitters, thereby decreasing spasticity [9]. Oral baclofen has limited use due to side effects, like sedation [1, 4]. Intrathecal baclofen was introduced as an alternative to oral baclofen in the 1990s. Baclofen is infused into the intrathecal space through a catheter connected to an implanted programmable pump [4, 10]. Intrathecal baclofen pumps (ITBPs) offer enhanced efficacy and fewer side effects because the formulation is administered locally in micrograms rather than milligrams [4, 10]. While there are several advantages to ITBPs, including improved pain management and gross motor function, they are associated with serious complications such as infection, withdrawal, and implant dysfunction [10–12].

Children with greater neurological involvement, especially those who are non-ambulatory, are at increased risk for both progressive scoliosis and severe spasticity [4]. Therefore, treatment plans often include both spinal deformity surgery for curve correction and an ITBP for tone management. The influence of ITBP on outcomes following spinal fusion surgery in patients with NMS is poorly understood. Case series and small retrospective studies have attempted to determine the risks associated with spine surgery for patients with ITBPs, but results are equivocal with some reporting higher rates of complications [13, 14] and others reporting similar outcomes between patients with and without ITBPs [15–18]. Two case reports described severe withdrawal from baclofen due to pump malfunctions occurring after surgery, and in one case, it led to the death of a patient [12, 19]. However, sample sizes for patients in the ITBP groups across all studies were relatively small, ranging from 5 to 53 participants [12–20], which may explain inconsistencies in findings.

Uncertainty about additional risks related to spinal deformity surgery for patients with ITBP presents challenges for caregivers who need to understand potential complications and risks their child may face. Thus, this study examined complications in children with NMS associated with CP and compared complication rates between those with and without ITBP. We hypothesized that patients with ITBP are at a higher risk for postoperative complications following spinal fusion surgery when compared to patients without ITBP.

## Methods

This retrospective cohort study was reviewed and approved by a University of Minnesota Institutional Review Board, and a waiver of consent was granted due to the retrospective nature of the study.

### Patients

Eligibility criteria were: (1) diagnosis of CP with associated scoliosis, (2) gross motor function classification system (GMFCS) levels IV–V, (3) index spinal fusion surgery before 21 years of age, (4) index surgery between 2001 and 2021, and (4) at least 2 years of follow-up. Patients with prior spinal deformity surgery (e.g., prior growing rod surgery), index surgery at a different site, anterior-only surgical approach, and those who opted out of research activities were excluded.

To identify eligible patients, an initial list was created using diagnosis codes for CP and NMS and procedure codes for spinal fusion. Medical documentation for these patients was then screened to confirm eligibility.

Patients were divided into two groups based on whether they had or did not have an ITBP at the time of their spinal fusion procedure. Spasticity/tone management is part of routine care provided by physical medicine and rehabilitation physicians at our institution, who provide care for most patients with non-ambulatory cerebral palsy (such as those in this sample). The decision of who gets a pump is based on a multidisciplinary discussion with the patient’s care team, including physical medicine and rehabilitation, neurosurgery, and the patient and family.

At our institution, baclofen pumps are recommended for patients with severe spasticity interfering with function, comfort, and/or care provision which has not responded adequately to oral or injected medications. After the recommendation is made, the patient/legally authorized decision maker has the option to decide whether to proceed with pump implantation. Not all who would be a good candidate for a pump choose to receive them.

### Procedure

Data for eligible patients were abstracted from the institution’s medical record into a Research Electronic Data Capture (REDCap) database (Vanderbilt University, Nashville, TN) [21, 22]. Clinical data abstracted for this study included demographic data, preoperative clinical history (e.g., type of CP, GMFCS level [23] and subclassification [24]), index surgery information (e.g., surgery date, length of procedure), and postoperative outcomes (e.g., complications, length of hospital stay). GMFCS classification by physicians at our institution was based on the original publication by Palisano et al., which defines GMFCS IV and V as relying mainly or solely on wheeled mobility at home, school, and in the community [23]. Perioperative and short-term (i.e., < 90 days after surgery) complications included durotomy, unplanned re-intubation, escalation of care, non-musculoskeletal infections treated with an antibiotic (e.g., pneumonia, urinary tract infection), superior mesenteric artery (SMA) syndrome, venous thromboembolic (VTE) disease, non-infectious pulmonary complications (e.g., pleural effusion requiring drainage), neurological complications, readmission, ITBP-related complications, and mortality. Longer term complications included reoperation related to the index surgery, including reasons for reoperation (e.g., pseudoarthrosis, surgical site infection (SSI)). Radiographic outcomes were measured by a single, trained evaluator. All radiographs were seated films. Preoperative films were the most recent films taken before the index surgery. Postoperative films were taken 3–12 weeks after the index surgery. Eleven orthopedic surgeons provided spine surgery at our institution during the period of this study, with years of experience ranging from 1 to 35 years of experience over this time. Neurosurgery providers were not typically involved in primary spine surgeries but were involved when there were ITBP-related procedures or complications.

### Analysis

Descriptive statistics were calculated for all variables across the total cohort and by study group. The distribution of all continuous outcomes was assessed for normality using histograms and quantile–quantile plots. As data were not normally distributed, medians were reported and non-parametric tests were used. Outcomes and complications were compared between patients with and without ITBP using Fisher’s exact and Mann–Whitney *U* tests. To assess whether there were pre-surgical differences in groups (e.g., that patients with ITBP had greater disease severity than those without ITBP), binomial logistic regression and multivariable linear regression models were used to assess confounding by demographic and baseline clinical characteristics (gender, age, GMFCS, Body Mass Index, preoperative curve magnitude, and year of index surgery), using the 10% rule of confounding. For all analyses, the significance threshold was defined as alpha<0.05. R statistical software was used for all analyses.

## Results

### Study sample

**Fig. 1:**
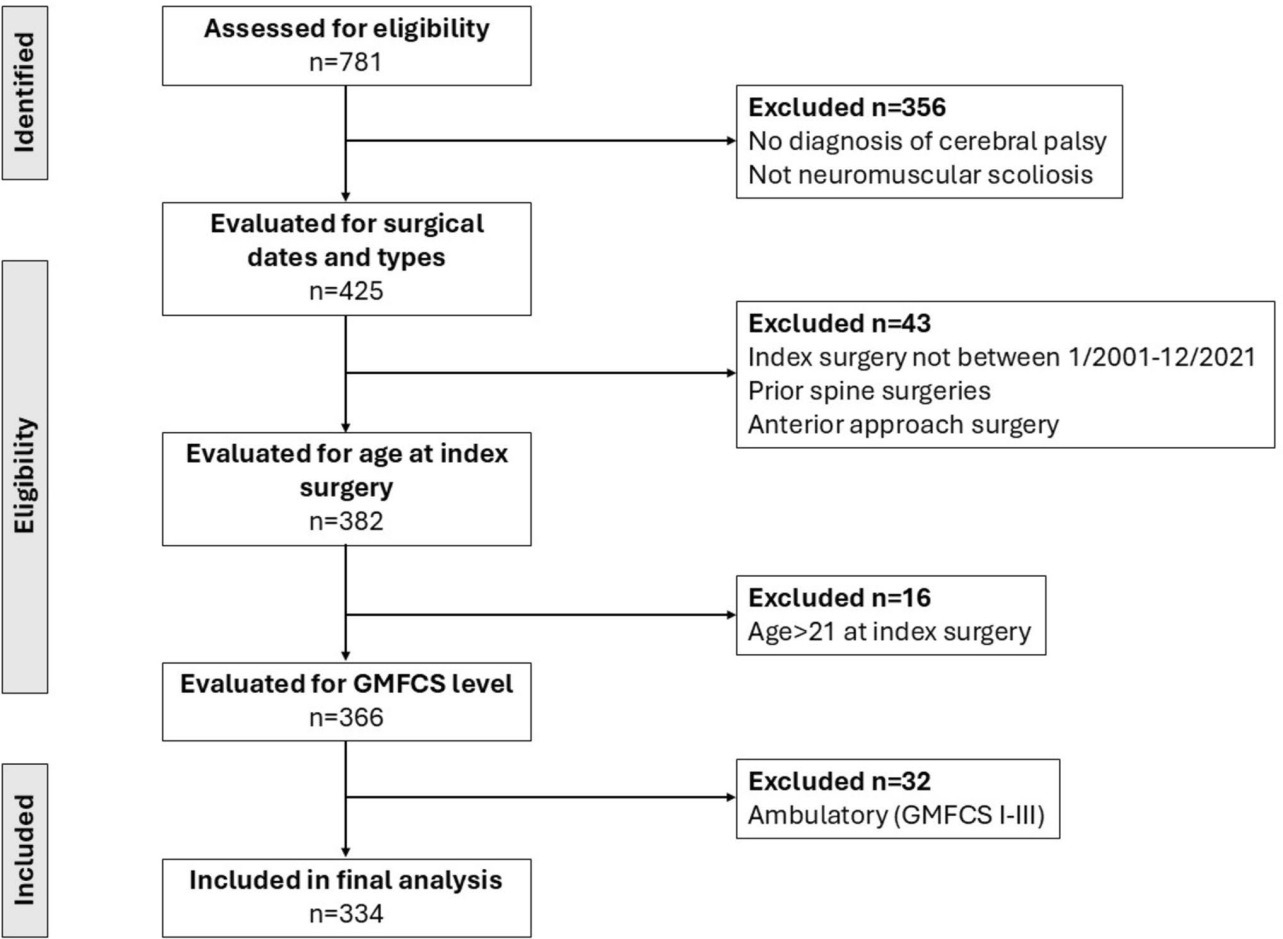
CONSORT flow diagram depicting patient eligibility for the study

A total of 334 patients were eligible (Fig. 1): 163 with ITBP, 171 without ITBP (mean age 14.0 ± 3.5 years, 59.6% male). Most were GMFCS V (73.1%) and the remaining were GMFCS IV (26.9%) (Table 1). Average follow-up time was 4.5 ± 4.8 years.

**Table 1:** Participant characteristics

	ITBP *N* = 163	No ITBP *N* = 171	Total sample *N* = 334
*Gender*			
Female	51 (31.3)	84 (49.1)	135 (40.4)
Male	112 (68.7)	87 (50.9)	199 (59.6)
Age (years)	14.3 (3.3)	13.6 (4.1)	14.0 (3.5)
*Race*			
White	133 (81.6)	121 (71.6)	254 (76.5)
Black or African American	17 (10.4)	25 (14.8)	42 (12.7)
Asian	2 (1.2)	5 (2.9)	7 (2.1)
American Indian/Alaska native	1 (0.6)	3 (1.8)	4 (1.2)
Other or declined	10 (6.1)	15 (8.9)	25 (7.5)
*Ethnicity*			
Hispanic	5 (3.1)	12 (7.0)	17 (5.1)
Not hispanic	145 (88.9)	149 (87.1)	294 (88.0)
Missing or declined	13 (8.0)	10 (5.9)	23 (6.9)
*CP type*			
Spastic	149 (91.4)	151 (89.3)	300 (90.4)
Dyskinetic	1 (0.6)	0 (0.0)	1 (0.3)
Ataxic	0 (0.0)	0 (0.0)	0 (0.0)
Mixed	13 (8.0)	18 (10.7)	31 (9.3)
*CP pattern*			
Diplegia	2 (1.2)	2 (1.2)	4 (1.2)
Hemiplegia	0 (0.0)	0 (0.0)	0 (0.0)
Triplegia	1 (0.6)	3 (1.8)	4 (1.2)
Quadriplegia	160 (98.2)	166 (97.0)	326 (97.6)
*GMFCS*			
IV	50 (30.7)	40 (23.4)	90 (26.9)
V	113 (69.3)	131 (76.6)	244 (73.1)
*GMFCS V subcategories*			
5.0	3 (2.8)	2 (1.6)	5 (2.2)
5.1	18 (17.0)	9 (7.3)	27 (11.8)
5.2	23 (21.7)	19 (15.5)	42 (18.3)
5.3	62 (58.5)	93 (75.6)	155 (67.7)
Pre-op curve magnitude	77.0 (32.8)	75.0 (29.5)	76.0 (31.4)
*Pre-op curve location*			
Thoracic	30 (18.4)	36 (21.2)	66 (19.8)
Thoracolumbar	60 (36.8)	69 (40.6)	129 (38.7)
Lumbar	73 (44.8)	65 (38.2)	138 (41.4)
*Surgical approach*			
Posterior	146 (89.6)	155 (90.6)	301 (90.1)
Anterior and posterior	17 (10.4)	16 (9.4)	33 (9.9)
*BMI percentile categories*			
Underweight	57 (37.0)	52 (31.9)	109 (34.4)
Normal weight	95 (61.7)	105 (64.4)	200 (63.1)
Obese	2 (1.3)	6 (3.7)	8 (2.5)
Follow-up time (years)	4.2 (5.2)	4.5 (4.6)	4.5 (4.8)

The total number of surgeries across surgeons during the study period was between 2 and 59 each, with an average of 30 (SD 19) per surgeon. Each surgeon performed ≤ 18% of the total surgeries, and there were five surgeons who performed between 12 and 18% of the total surgeries.

### Complications and other clinical outcomes

Patients with ITBP were not more likely to experience complications compared to those without ITBP (*p* = 0.19, Table 2). However, those with ITBP did have statistically significantly greater odds of SSI (ITBP: 8.6%; no ITBP: 2.9%, OR: 3.11, *p* = 0.03), longer median surgery duration (ITBP: 444 min; no ITBP: 391 min, *p* < 0.001), and higher median percentage of blood loss during surgery, measured as a percent of total volume (ITBP: 36.3%; no ITBP: 27.2%, *p* = 0.01). There were no other statistically significant differences in rates of individual complications or post-op measures between groups. Demographic and clinical characteristics, including year of surgery, were found not to confound the relationship between having an ITBP and complications/clinical outcomes.

**Table 2:** Complication and perioperative outcomes for patients with CP and neuromuscular scoliosis in the sample (*n* = 334). Data are presented for those with (*n* = 163) and without (*n* = 171) intrathecal baclofen pumps (ITBP)

	ITBP	No ITBP	OR (95% CI)	*p*-value
	*n* = 163	*n* = 171		
*Complications*				
At least one complication	88 (54.0%)	79 (46.2%)	1.37 (0.87, 2.15)	0.19
*Individual complications*				
Durotomy	5 (3.1%)	4 (2.3%)	1.32 (0.28, 6.78)	0.75
Unplanned intubation	7 (4.3%)	6 (3.5%)	1.23 (0.35, 4.55)	0.78
Escalation of care	12 (7.4%)	20 (11.7%)	0.60 (0.26, 1.34)	0.20
Non-musculoskeletal infection treated with antibiotics (e.g., pneumonia, UTI)	35 (21.5%)	32 (18.7%)	1.19 (0.67, 2.11)	0.59
Superior mesenteric artery syndrome	2 (1.2%)	2 (1.2%)	1.05 (0.08, 14.63)	1
Venous thromboembolism	1 (0.6%)	1 (0.6%)	1.05 (0.01, 82.80)	1
Non-infectious pulmonary complications (e.g., symptomatic pleural effusion)	3 (1.8%)	7 (4.1%)	0.44 (0.07, 1.97)	0.34
Neurological complications (*N* = 333)	3 (1.9%)	2 (1.2%)	1.59 (0.18, 19.29)	0.68
Readmission	27 (16.6%)	22 (12.9%)	1.34 (0.70, 2.60)	0.36
Mortality	2 (1.2%)	1 (0.6%)	2.11 (0.11, 125.22)	0.62
Reoperation	34 (20.9%)	24 (14.0%)	1.61 (0.88, 3.00)	0.11
Reason for reoperation***				
Pseudoarthrosis	12 (7.4%)	11 (6.4%)	1.16 (0.45, 2.99)	0.83
Wound infection	14 (8.6%)	5 (2.9%)	3.11 (1.03, 11.30)	0.03*
Implant misplacement	2 (1.2%)	1 (0.6%)	2.11 (0.11, 125.22)	0.62
Proximal junctional kyphosis	3 (1.8%)	2 (1.2%)	1.58 (0.18, 19.17)	0.68
Distal curve progression	1 (0.6%)	2 (1.2%)	0.52 (0.01, 10.13)	1
Prominent/symptomatic implants	5 (3.1%)	6 (3.5%)	0.87 (0.21, 3.50)	1
Acute implant failure	0 (0.0%)	1 (0.6%)	–	1
Other causes	2 (1.2%)	0 (0.0%)	–	0.24
ITB-related complications	18 (11.0%)	N/A	–	
*Perioperative characteristics*				
Length of ICU stay, days (*N* = 302)	2.0 (2.0)	2.0 (1.0)	–	0.55
Length of hospital stay, days	7.0 (3.0)	7.0 (3.0)	–	0.30
Post-op curve magnitude, degrees (N=330)	32.1 (24.8)	30.2 (23.0)	–	
Curve correction, % (*N* = 331)	60.8 (31.8)	60.0 (26.3)	–	0.92
Duration of surgery, minutes (*N* = 250)	444 (148.8)	391 (157.5)	56.0 (25.0, 84.0)**	**< 0**.001*
Blood loss, % (*N* = 307)	36.3 (36.3)	27.2 (26.0)	6.1 (1.7, 11.0)**	**0**.01*
Required transfusion during procedure (*N* = 296)	72 (50.7%)	67 (43.5%)	1.33 (0.82, 2.17)	0.24

Values represent *n* (%) for categorical variables and median (IQR) for continuous variables

*Denotes statistical significance at alpha < 0.05

**Test statistic represents median difference and 95% CI (for continuous data) instead of odds ratio

***Can have more than one reason for reoperation

Three children died within 90 days of index surgery (ITBP = 2 and non-ITBP = 1). The child without ITBP died of a sudden cardiac event 6 weeks postoperatively (4 weeks after being discharged from the hospital). She had several severe comorbidities, including restrictive lung disease, seizures, a known heart murmur and obstructive sleep apnea treated with continuous positive airway pressure (CPAP). One child with an ITBP had experienced significant blood loss (1300 mL) during surgery but had an uncomplicated postoperative period. He had comorbidities including sleep apnea treated with CPAP and history of seizures as an infant. On the fifth postoperative day, he was discharged as his pain was well controlled and he was hemodynamically stable. The next day, his mother called to report that he had died in his sleep. The exact cause is unknown, and we were unable to determine if this death was related in any way to the ITBP. The other child with ITBP had a complicated postoperative course. On postoperative day five, a sepsis workup was initiated due to somnolence and being febrile. His baclofen dose was also decreased. It was determined that his intravenous line (IV) was infected with coagulase-negative Staphylococcus. He was treated with vancomycin and cefotaxime. Then, on postoperative day eight, a dural tear and pseudomeningocele were discovered and treated surgically, during which the catheter was inspected, but no obvious holes or tears were observed. On postoperative day 17, due to severe agitation days prior, baclofen withdrawal and rhabdomyolysis were diagnosed (CPK level was 120,000 U/L). He was treated with hydration, urine alkalinization, oral baclofen in addition to intrathecal baclofen boluses every 6 h. This initiated a discussion of palliative care. As care progressed, the pain became intractable, and palliative care was agreed upon on postoperative day 32. The child died on postoperative day 35. Notes prior to his death indicated a modified Ashworth score of 1-2 and no clonus, which indicates that his baclofen withdrawal was adequately treated at the time of death.

### Baclofen pump-related complications

Of patients with ITBPs, 11.0% (*n* = 18) had at least one pump-related complication in the intraoperative (*n* = 11) and/or postoperative (*n* = 14) periods (Table 3, Online Appendix 1). Of the 11 patients with intraoperative complications, most involved the catheter and were resolved by catheter revision (e.g., replacement or splicing) by a pediatric neurosurgeon during the index surgery (*n* = 8). Of the 14 patients with pump-related complications in the first 90 days after surgery, most had ITBP and/or catheter infection secondary to deep SSI (*n* = 8) that was resolved by pump and/or catheter removal (*n* = 8). One patient in our sample experienced a cerebral spinal fluid leak (CSF). This patient had two separate catheters at the time of the fusion, one connected and one abandoned. The neurosurgery team was consulted, and the abandoned catheter was removed from the dural space during the spinal fusion to reduce risk of infection. One month after the procedure, the patient exhibited signs of a low-pressure CSF leak. Symptoms were managed conservatively for 6 months without success. Ultimately, the pump and catheter were replaced and symptoms resolved.

**Table 3:** Intrathecal baclofen pump-related complications and outcomes

	ITBP (*n* = 163)	
	*N*	%
Total number of patients with pump-related complications	18	11.0
Number of patients with intraoperative pump-related complication	11	6.7
Intraoperative pump-related type*		
Catheter revision (replacement, splicing)	8	4.9
Inadvertent sectioning of catheter	4	2.5
Catheter obstruction	3	1.8
Inadvertent dislodgement from intrathecal space	3	1.8
Signs of temporary baclofen withdrawal	1	0.6
Number of patients with postoperative pump-related complications	14	8.6
Post-op pump-related complication type*		
Pump and/or catheter removal	8	4.9
ITB pump and/or catheter infection (secondary to deep SSI)	8	4.9
Signs of temporary baclofen withdrawal	4	2.5
Catheter obstruction	3	1.8
Pump and catheter replacement	2	1.2
Acute baclofen withdrawal	1	0.6
Signs of baclofen overdose	1	0.6
Cerebral spinal fluid leak	1	0.6
ITB complication led to readmission	11	6.7
Additional surgical intervention (after index procedure)	11	6.7

*Can have more than one type of complication

For surgeries with patients who have ITBP (*n* = 163 patients), individual surgeons performed ≤ 19% of the surgeries (1–31 surgeries per surgeon), and there were four surgeons who each performed between 13 and 19% of surgeries. Across surgeons, the number of complications ranged from 0 to 4 ITBP-related complications per surgeon. Complication rates ranged between 0% and 20%, with an average of 10% (SD 6%) ITBP-related complications across surgeons. For surgeons who conducted over 13% of the surgeries in patients with ITBP (four surgeons), the ITBP-related complication rates ranged from 8 to 14%. One surgeon on the team intentionally cut the catheter. This surgeon conducted 13% of the surgeries for patients with ITBP and had 3 ITBP-related complications (ITBP-related complication rate of 14%), with no patterns on type of ITBP-related complication. Others who conducted larger proportions (> 13%) of surgeries in children with ITB pumps had between 2 and 4 ITB-related complications.

## Discussion

Our study demonstrates that non-ambulatory children with CP who have ITBPs are not at increased risk of perioperative complications when undergoing spinal fusion surgery compared to those without ITBPs. The overall proportion of patients who experienced at least one complication was higher in the ITBP cohort, but this finding was not statistically significant. The only individual complication that significantly differed between groups was SSI, which was about three times more likely to occur in patients with ITBP. There were no other individual complications that were more likely to be experienced in one cohort versus the other, even though 11% of patients in the ITBP cohort had an ITBP and/or catheter-related complication.

Other retrospective studies have investigated complication rates between children with and without ITBP, with mixed conclusions. Similar to the present study, Yaszay et al. and Levy et al. found no difference in complications between those with an ITBP and those without an ITBP [15, 18] In contrast, Buxton et al. found a significant difference between groups, but the cohort without an ITBP had a higher complication rate [16]. Study authors did not comment on the clinical underpinnings for this unique finding. Caird et al. found a significant difference in the reoperation and readmission rates between ITBP and non-ITBP cohorts (total *n* = 40). In their ITBP cohort, 45% (9/20) underwent reoperation, whereas 20% (4/20) in the non-ITBP cohort required reoperation [13]. These percentages were higher than those in our study, where 21% (34/163) of children with an ITBP and 14% (24/171) of children without an ITBP underwent reoperation. Caird et al. also reported a significant difference in readmission; 40% (8/20) of their ITBP cohort was readmitted compared to 10% (2/20) in the non-ITBP cohort. Readmission in our study occurred in 17% (27/163) and 13% (22/171) in the ITBP and non-ITBP cohorts, respectively. The notable discrepancies in readmission and reoperation rates between these two studies may be due to differences in sample sizes. In the study conducted by Caird et al., each cohort had a sample size of 20, whereas there were more than 160 in each of our cohorts. This present study is the largest series to date and, therefore, is a valuable contribution to the presently equivocal body of evidence.

While there were no significant differences in overall reoperation rates, there were eight children who experienced pump- and/or catheter infections that required reoperation, which contributes to the higher odds of SSI in patients with ITBP found in this study. Xu et al. [25] also found a high rate of wound infections in patients with neuromuscular scoliosis who had ITBP (*n* = 25), reporting that patients with ITBP had 4.7 times the odds of deep SSI compared to controls without ITBP. Borowski et al. [17] studied children with CP undergoing spinal fusion and compared complication rates based on when the ITBP was placed (before, during, or after surgery) versus children who had an ITBP implanted but did not have spinal deformity surgery. Study investigators did not find differences in infection rates between groups, and also reported infection and complications in patients with ITBP who did not undergo spinal fusion. Of the participants who did *not* undergo spinal fusion, 27% experienced a device-related complication and 9% developed an infection. In the cohort where an ITBP was placed prior to surgery, 16% experienced a device-related complication and 8% developed an infection [17]. It is difficult to make a definitive conclusion due to the small samples in each cohort (*n* = 11–26), but this study showed that ITBPs themselves carry an increased risk for independent device-related complications and infection not experienced by those without ITBP. The presence of ITBP presents additional difficulties during spine deformity surgery, including technical challenges related to achieving a water-tight fascial closure around the catheter while avoiding puncture of the catheter tip with suture or needle material. In addition, the higher risk of infection in the ITBP patient group could be related to the greater blood loss and operative time found in this study and the presence of additional prosthetic material (e.g., the catheter tip) in the superficial region of the operative site. Lastly, there may be a small increased risk of infection in this ITBP patient cohort related to wound healing challenges through an area of prior scar. In our study, 11% of children with ITBP had an ITBP-related complication and ITBP-related complications led to readmission to the hospital in 6.7% of children, who all needed additional surgical intervention to address an ITBP-related complication. Eight children had their pump and/or catheter removed due to deep SSI related to their spinal fusion. Removal in each case was recommended by the pediatric infectious disease service who felt that colonization of the baclofen pump in direct communication with central nervous system/CSF carried unacceptable risk. While the percentages are small for each scenario, it is important to acknowledge the risks, particularly in discussions with children and their caregivers.

Two perioperative characteristics significantly differed in the ITBP compared to the non-ITBP cohorts in our study: duration of surgery and estimated blood loss percentage. We speculate that this may reflect the greater complexity of operating around the catheter, though the higher estimated percent blood loss could potentially be due to other factors, such as differences in medications or medical fragility between groups. Caird et al., Buxton et al., and Yaszay et al. found that the mean blood loss was higher in the ITBP cohort, but this difference was not statistically significant [13, 16, 18]. Interestingly, Yaszay et al.’s non-ITBP cohort had a higher mean total operating time [18]. This is unlike Caird et al.’s and Buxton et al.’s studies, where the ITBP cohorts experienced a higher mean of total operating time [13, 16]. However, none of these results were statistically significant, most likely due to small sample sizes, which may have led to underpowered analyses. In addition, non-ambulatory patients with CP have comorbidities and undergo various additional treatments, which may have contributed to the discordant results reported across studies.

What is consistently seen across studies is that children with CP, whether they have ITBP or not, have higher complication rates than other populations who undergo spinal deformity surgery. At our institution, all children with CP who undergo spine surgery are evaluated by our Presurgical Patient Assessment and Risk Evaluation (PrePARE) team because of the higher risk of complications. Our PrePARE team is composed of multidisciplinary providers, including anesthesiologists and complex care pediatricians. In addition, each child’s case is discussed at a monthly multidisciplinary spine conference, which is attended by the PrePARE team and orthopedic spine providers. This additional attention to the care of medically fragile patients ensures optimization of health prior to undergoing spinal deformity surgery.

To our knowledge, this is the first study to publish an ITBP cohort of this magnitude. However, it is not without limitations. This study was retrospective and all the data were pulled from a single center. These data span 20 years, and in that time, surgical techniques, perioperative medical care, and implants have changed, but similar percentages of patients in both cohorts were seen across the 20-year period. At our institution, there was no standardized approach for management of the catheter during the procedure. One surgeon often intentionally cut the catheter and then repaired it after the instrumentation was placed, while other surgeons operated around the catheter. We were not able to reliably abstract information about catheter management for each patient in this study, and thus we are unable to compare outcomes between the techniques of intentionally cutting the catheter and working around the catheter in this study. Note that intentional cutting of the catheter was not included as an ITBP complication in this study. Lastly, in our analyses, some outcomes had very small cell sizes (n ≤ 5), therefore lacking the power to detect significant differences in individual complications.

## Conclusion

Children with CP who have ITBP were not more likely to experience complications following spine fusion surgery compared to those without ITBP. However, children with ITBP had a greater risk for SSI, longer surgery durations, and a higher percentage of blood loss. In addition, 11% of children who had ITBP developed a perioperative ITBP-related complication. Results will help improve counseling between surgeons, patients, and caregivers related to the risks of spinal fusion surgery when ITBPs are present.

## Supplementary Information

Below is the link to the electronic supplementary material.

Supplementary file1 (DOCX 93 kb)

## Data Availability

De-identified data will be made available upon reasonable written request to the corresponding author.
