# Supplementary material for: The influence of intrathecal baclofen pumps on outcomes following spinal fusion in non-ambulatory patients with cerebral palsy
Source: Spine Deform. 2026 Jan 17;14(3):999–1008. doi: 10.1007/s43390-026-01274-5 (PMC13282316; doi:10.1007/s43390-026-01274-5)
Supplement: Supplementary file 1 — Supplementary file1 (DOCX 93 kb) [file 43390_2026_1274_MOESM1_ESM.docx]

Appendix 1. Individual patient information about pump-related complications.

| **Patient**  **ID** | **Patient characteristics** | | | **Curve magnitude** | | | **Index fusion** | | | **Pump-related complications** | | **Additional surgery required** | **Resolution of complication** |
| --- | --- | --- | --- | --- | --- | --- | --- | --- | --- | --- | --- | --- | --- |
|  | **Age/sex** | **GMFCS** | **Curve loc.** | **Pre-op** | **Post-op** | **% corr.** | **Surgery duration (min)** | **% blood loss** | **Surg approach** | **Intra-op** | **Post-op** |  |  |
| 1 | 10 y/o Female | 5.1 | T | 81˚ | 3˚ | 96% | 416 | 9% | Post | None | Catheter obstruction  Signs of baclofen withdrawal | No | Bolus of ITB |
| 2 | 10 y/o Male | 5.3 | T | 75˚ | 34˚ | 54% | 457 | 27% | Post | None | ITB pump infection (secondary to deep SSI) | Yes- readmitted | Reoperation to address SSI, pump and catheter removal |
| 3 | 10 y/o Male | 5.2 | T | 65˚ | 11˚ | 83% | 369 | 21% | Post | None | Signs of baclofen withdrawal  ITB pump infection (secondary to deep SSI) | Yes- readmitted | Reoperation to address SSI, pump and catheter removal |
| 4 | 15 y/o Female | 5.1 | TL | 86˚ | 28˚ | 67% | 638 | 26% | Post | Catheter obstruction | Catheter obstruction | Yes- readmitted | Intraoperative revision of the catheter (at index procedure); additional surgery post-operative for revision of the catheter |
| 5 | 15 y/o Male | 4 | TL | 128˚ | 46˚ | 64% | Missing data | 45% | Post | Inadvertent sectioning of the catheter | None | No | Intraoperative revision of the catheter (at index procedure) |
| 6 | 14 y/o Female | 4 | TL | 65˚ | 19˚ | 71% | 512 | 53% | Post | Inadvertent sectioning of the catheter | ITB catheter infection (secondary to deep SSI) | Yes- readmitted | Intraoperative revision of the catheter (at index procedure); Reoperation to address SSI, catheter removal |
| 7 | 8 y/o Male | 5.3 | TL | 59˚ | 14˚ | 76% | Missing data | 122% | Post | Inadvertent dislodgement from intrathecal space | ITB pump infection (secondary to deep SSI) | Yes- readmitted | Intraoperative revision of the catheter (at index procedure); Reoperation to address SSI, pump and catheter removal |
| 8 | 16 y/o Male | 4 | TL | 66˚ | 25˚ | 62% | 603 | 45% | Post | None | Cerebral spinal fluid leak | Yes- readmitted | Conservative management with bed rest and Diamox, ultimately the pump was removed and replaced |
| 9 | 14 y/o Male | 5.1 | TL | 100˚ | 36˚ | 64% | 501 | 81% | Post | Inadvertent dislodgement from intrathecal space | None | No | Intraoperative revision of the catheter (at index procedure) |
| 10 | 13 y/o Male | 5.3 | L | 55˚ | 2˚ | 96% | 420 | 19% | Post | Catheter obstruction  Signs of temporary withdrawal | ITB pump and catheter infection (secondary to deep SSI) | Yes- readmitted | Bolus of ITB;  Reoperation to address SSI, pump and catheter removal |
| 11 | 12 y/o Female | 5.3 | L | 93˚ | 47˚ | 50% | 356 | 41% | Post | Catheter obstruction (catheter stuck and could not be reinserted during fusion surgery) | None | Yes- additional surgery while inpatient | Surgical removal of bone required to restore catheter function during index surgery |
| 12 | 13 y/o Male | 5.1 | L | 56˚ | 17˚ | 70% | Missing data | 50% | Post | ITB catheter obstructing fusion surgery | Catheter obstruction  Signs of baclofen withdrawal  Acute baclofen withdrawal | Yes- additional surgery while inpatient and also readmitted | Cut the rod and connected the segments to pass the rod under the catheter during index procedure; additional surgery post-operative for pump replacement |
| 13 | 13 y/o Male | 5.3 | L | 72˚ | 17˚ | 76% | 339 | 48% | Post | Inadvertent sectioning of the catheter | ITB pump and catheter infection (secondary to SSI) | Yes- readmitted | Intraoperative revision of the catheter (at index procedure); Reoperation to address SSI, pump and catheter removal |
| 14 | 15 y/o Male | 4 | L | 93˚ | 68˚ | 27% | 529 | 27% | Post | Inadvertent sectioning of catheter | Signs of baclofen overdose | No | Intraoperative revision of the catheter (at index procedure); adjusted ITB dosage to address signs of overdose |
| 15 | 14 y/o Male | 5.1 | L | 73˚ | 39˚ | 47% | Missing data | Missing data | Post | Inadvertent dislodgement from intrathecal space | None | No | Intraoperative revision of the catheter (at index procedure) |
| 16 | 13 y/o Male | 5.3 | L | 74˚ | 58˚ | 22% | 651 | 23% | Ant and post | None | ITB catheter infection (secondary to deep SSI) | Yes- readmitted | Reoperation to address SSI, pump and catheter removal |
| 17 | 19 y/o Male | 5.1 | L | 102˚ | 49˚ | 52% | 666 | 44% | Ant and post | None | ITB pump infection (secondary to SSI) | Yes- readmitted | Reoperation to address SSI, pump and catheter removal |
| 18 | 11 y/o Male | 5.3 | TL | 69˚ | 19˚ | 72% | 384 | 39% | Post | None | Signs of baclofen withdrawal | No | Periodic ITB bolus dosing and oral baclofen, patient died of pneumonia in the post-operative period |
